# The effects of stroboscopic vision training on sports vision ability performance and punching performance in female boxers

**DOI:** 10.3389/fphys.2026.1856441

**Published:** 2026-06-10

**Authors:** Youlin Xiao, Dongxu Gao, Mingnan Zhuang, Yuankun Long, Qiang Wei, Dexin Wang, Peng Zhang, Zihao Zhao, Qiangquan Wang, Xinbo Zhang

**Affiliations:** 1 Sports College, Dalian University, Dalian, China; 2 Tsinghua University, Beijing, China; 3 Tianjin University of Sport, Tianjin, China; 4 Shanghai University of Sport, Shanghai, China; 5 Jiamusi University, Jiamusi, China; 6 Shaanxi University of International Trade, XiAn, China; 7 Ningxia Normal University, Yuanzhou Qu, China

**Keywords:** boxing performance, female boxers, perception-cognition, sports vision ability, stroboscopic training

## Abstract

**Background:**

Stroboscopic training uses intermittent visual occlusion to disrupt continuous visual information flow, inducing adaptive improvements in visuomotor processing. Although its efficacy has been demonstrated across several fast-response sports, its application in boxing — particularly among female athletes — remains largely unexplored. This study investigated the effects of a six-week stroboscopic training program on visual performance skills and punching accuracy in female amateur (Olympic-style) boxers, and examined the durability of training effects at a four-week follow-up.

**Methods:**

Twenty-six female amateur boxers (mean age 24.69 ± 5.48 years; mean boxing experience 7.19 ± 2.51 years) were randomly assigned to a stroboscopic training group (n = 13) or a non-stroboscopic control group (n = 13). Visual performance was assessed across 10 metrics using the Senaptec Sensory Station at pre-test, post-test, and retention. Punching accuracy (%Hit) was assessed from video recordings of official bouts and formal intra-team sparring sessions. The six-week intervention consisted of three 35-minute sessions per week combining general visual reaction drills and boxing-specific reactive tasks, performed under stroboscopic or normal visual conditions.

**Results:**

Significant group × assessment phase interactions were observed for five visual performance metrics: Eye-Hand Coordination (EHC; F(2,48) = 6.105, p = 0.004, ηp² = 0.203), Reaction Time (RT; F(2,48) = 3.230, p = 0.048, ηp² = 0.119), Go/No-Go (GNG; F(2,48) = 4.807, p = 0.013, ηp² = 0.167), Perception Span (PS; F(2,48) = 6.005, p = 0.005, ηp² = 0.200), and Multiple Object Tracking (MOT; F(2,48) = 6.039, p = 0.005, ηp² = 0.201). Punching accuracy also improved significantly (F(2,48) = 4.626, p = 0.015, ηp² = 0.162), with post-test %Hit (35.02 ± 7.27%) significantly higher than pre-test (28.85 ± 7.45%). Training effects on EHC, GNG, PS, and MOT were partially retained at the four-week follow-up; RT and %Hit improvements were not retained. No significant changes were observed for basic visual functions (VC, CS, DP, TC, NFQ) in either group.

**Conclusions:**

Six weeks of stroboscopic training significantly improved key visuomotor skills and punching accuracy in female amateur boxers, with partial retention of effects four weeks post-intervention. These findings support the integration of stroboscopic training into evidence-based boxing preparation programs and highlight the need for periodic booster sessions to sustain long-term gains.

## 1 Introduction

### 1.1 Visual performance skills in boxing

Visual performance skills represent a multifaceted integration of sensory perception, neural processing, and motor response coordination—capabilities that are not merely supplementary but foundational to elite performance in combat sports, especially boxing (Müller et al., 2024; Tarasi et al., 2024). Unlike static visual tasks (e.g., reading or identifying stationary objects) that rely on sustained focus on unchanging stimuli, boxing imposes extreme dynamic visual demands: athletes must simultaneously track an opponent’s limb movements, facial cues, and body posture; anticipate the trajectory and force of incoming punches; calibrate spatial distance between themselves and their opponent; and initiate precise, timed motor responses—all within a time window as short as 100–300 milliseconds for defensive reactions and 402–405 milliseconds for offensive punch delivery (Stanley et al., 2018). This high-stakes, time-constrained environment elevates visual performance to a primary differentiator between successful and unsuccessful boxers, as even a 50-millisecond delay in visual processing can result in a missed defensive opportunity or an inaccurate punch.

Key dimensions of visual performance that are particularly salient to boxing include eye-hand coordination (the ability to synchronize visual input with manual motor actions), rapid reaction time (the latency between visual stimulus detection and motor initiation), selective attention (the capacity to focus on relevant cues—e.g., an opponent’s jab—while filtering out distractions), multiple object tracking (monitoring multiple moving targets, such as an opponent’s hands and feet), and near-far quickness (rapidly shifting focus between close-range targets, like a focus mitt, and distant targets, like an opponent’s torso) (Buscemi et al., 2024; Lee et al., 2022b; Millard et al., 2020);. Epidemiological and sports science research has consistently confirmed the primacy of vision in athletic decision-making: approximately 80% of the sensory information athletes use to navigate competitive environments and execute tactical choices is derived from the visual system (Ripoll et al., 1995). In boxing, this statistic is amplified, as the sport’s confrontational nature leaves no margin for error in interpreting visual cues—every misjudgment of distance, timing, or opponent intent can lead to a competitive disadvantage or injury.

Elite boxers exhibit measurable superiority in visual performance metrics compared to sub-elite athletes and non-athletes, underscoring the role of visual skill in athletic development. For example, a study by Laby et al. (2011) demonstrated that Olympic-level boxers exhibited significantly superior stereoacuity (a critical component of depth perception) compared to other athletes, enabling them to more accurately estimate punching range during both close-quarters clinches and long-range striking exchanges. This precision is particularly valuable in modern boxing, where scoring criteria prioritize technical accuracy over brute force. Thomson et al., (2013) adoption of the “10-point must” scoring system marked a paradigm shift in bout evaluation, reorienting judges’ focus toward four core criteria: effective punching, defensive proficiency, ring generalship, and clean punching (Thomson et al., 2013). Among these, effective punching—defined as the number of legal punches landed on valid target areas (head, torso above the waist, and shoulders)—carries the highest weighting, accounting for approximately 40% of a judge’s scoring decision (Davis et al., 2013).

This scoring reform has disproportionately elevated the importance of punching accuracy (calculated as the percentage of successful punches relative to total punches thrown) in women’s boxing. Unlike men’s boxing, where knockout power is often a decisive factor, women’s boxing typically emphasizes technical precision, tactical awareness, and consistent punch placement—traits that are directly linked to visual performance. Dunn et al. (2017) further validated this relationship in a longitudinal study of 52 amateur female boxers, finding that athletes who landed 15% more scoring punches than their opponents had a 78% higher win rate in regional and national competitions. As a result, female boxers and their coaching staff have increasingly prioritized evidence-based visual training interventions as a means of improving punching accuracy, with targeted visual skill development emerging as a critical gap in current training paradigms.

### 1.2 Visual training in combat sports

Although targeted visual training has been proven to have significant efficacy, research on visual training in boxing has developed relatively slowly, and its overall effectiveness remains insufficient. Conventional visual training approaches typically include static eye exercises (e.g., eye rolls, focus on stationary objects), simple light reaction drills (e.g., responding to a single flashing light), and shadowboxing with static focus mitts. While these methods can produce modest improvements in basic visual skills (e.g., static focus), they lack the realism and complexity of actual competitive conditions. For instance, static eye exercises do not replicate the dynamic, fast-paced visual stimuli athletes encounter in the ring, and simple light drills fail to simulate the multi-cue environment of a boxing match (e.g., simultaneous movement of an opponent’s hands, feet, and body) (Appelbaum and Erickson, 2018). This disconnect limits the transferability of training effects to in-ring performance, as athletes are not challenged to apply their visual skills in contextually relevant scenarios.

A growing body of empirical research has established robust correlations between visual performance skills and striking performance in boxing, confirming that visual skill is not an innate trait but a malleable ability that can be enhanced through targeted training (Wu et al., 2024; Appelbaum et al., 2016). Wu et al. (2024), in a landmark study of 38 male amateur boxers, found that eye-hand coordination (r=0.62, p<0.001), reaction time (r=-0.58, p<0.001), and visual tracking ability (r=0.55, p<0.001) were strongly correlated with punching accuracy, indicating that improvements in these visual metrics could directly translate to better in-ring performance. Importantly, these correlations were not limited to male athletes: a follow-up study by the same research team, focusing on 29 female amateur boxers, reported similar effect sizes (r=0.59 for eye-hand coordination, r=-0.54 for reaction time), confirming the generalizability of the relationship between visual skill and punching accuracy across genders.

In recent years, technological advancements have paved the way for innovative visual training methods that address the limitations of traditional approaches, including prismatic adaptation (Giustino et al., 2024) and stroboscopic training. Among these, stroboscopic training has emerged as one of the most promising interventions, with a growing body of research demonstrating its efficacy in enhancing visual performance and sport-specific skills across a range of fast-response sports, including badminton, baseball, and volleyball (Appelbaum et al., 2016; Appelbaum and Erickson, 2018; Hülsdünker et al., 2019). Stroboscopic training differs from traditional visual training in its ability to simulate the dynamic, high-pressure visual environment of boxing by using intermittent visual occlusion (via specialized glasses) to disrupt the continuous flow of visual information. This “visual stress” induces adaptive changes in the nervous system, enhancing the brain’s ability to process visual cues quickly and accurately—skills that are directly applicable to boxing’s demands. Unlike traditional methods, stroboscopic training is inherently contextually relevant, as it requires athletes to apply visual skills in dynamic, movement-based tasks, thereby maximizing the transfer of training effects to in-ring performance.

### 1.3 Stroboscopic training: mechanisms and efficacy

The underlying mechanism of stroboscopic training is analogous to high-altitude training for endurance athletes (Appelbaum and Erickson, 2018). By creating a “stressful” visual environment (intermittent occlusion), the training induces adaptive changes in the nervous system that enhance performance under normal visual conditions. Specifically, stroboscopic stimulation has been shown to improve rapid information encoding, central and peripheral visual sensitivity, temporal anticipation, and functional connectivity between key brain regions involved in visual processing and motor control (Appelbaum et al., 2011, 2012; Lee et al., 2022a). Neuroimaging studies have further revealed that stroboscopic training enhances functional connectivity between the superior parietal lobule (SPL)—which may be important for spatial attention and visual-motor integration—and the premotor cortex (PMC)—which is involved in planning and executing motor responses (Ellison et al., 2020). This strengthened connectivity shortens the delay between visual input and motor initiation, a key advantage in fast-response sports like boxing.

A growing body of research has demonstrated the efficacy of stroboscopic training in improving sport-specific performance across various disciplines. Hülsdünker et al. (2019), for example, investigated the effects of a four-week stroboscopic training program in top-level badminton players, finding significant improvements in visual reaction time (reduced by 12 ms) and shuttlecock tracking accuracy (increased by 22%). Similarly, Liu et al. (2020) reported that a six-week dynamic vision training program (incorporating stroboscopic training) significantly improved launch angle and hit distance during batting practice among collegiate baseball players, further supporting the potential transfer effect of stroboscopic training to sport-specific batting performance (Liu et al., 2020). Other studies have shown positive effects in volleyball (improved reactive agility and saccadic eye movement speed (Zwierko et al., 2023)), soccer (enhanced dribbling accuracy and visual search efficiency (Palmer et al., 2022)), and softball (increased hitting accuracy and decision-making speed (Dendy et al., 2025)). These findings suggest that stroboscopic training is a versatile intervention that can be adapted to the specific visual demands of different sports.

Despite its proven efficacy in other fast-response sports, stroboscopic training has not been systematically investigated in boxing. This represents a significant gap in the literature, given the sport’s heavy reliance on visual performance skills. Hülsdünker et al. (2021) explicitly noted the need for validating stroboscopic training effects in sports beyond badminton, highlighting boxing as a compelling candidate due to its high visual-motor demands. Additionally, Wu et al. (2024) called for further research exploring how targeted visual training might enhance boxing performance, particularly in female athletes, who have been underrepresented in previous studies.

### 1.4 Rationale and research hypotheses

The primary rationale for this study is to address the gap in the literature by investigating the effects of stroboscopic training on visual performance skills and punching accuracy in female amateur boxers. Female boxers face unique physiological and competitive challenges (e.g., smaller body mass, different weight class distributions, and distinct scoring tendencies), making it inappropriate to generalize findings from male cohorts. Additionally, the study explores the retention of training effects four weeks post-intervention, a critical question for practical application (i.e., whether improvements are sustained beyond the training period). The present study therefore pursues three primary aims: (1) to determine whether a six-week stroboscopic training program improves key visual performance skills (EHC, RT, GNG, PS, MOT) in female amateur boxers compared to a control group; (2) to examine whether stroboscopic training enhances punching accuracy (%Hit) in official competitive settings; and (3) to assess the durability of any observed training effects at a four-week follow-up retention test.

Based on the existing literature, we formulated three primary research hypotheses:

Hypothesis 1: A six-week stroboscopic training program will significantly improve key visual performance skills (eye-hand coordination, reaction time, Go/No-Go, Perception Span, and Multiple Object Tracking) in female amateur boxers relative to a control group receiving non-stroboscopic training.

Hypothesis 2: The stroboscopic training program will significantly improve punching accuracy (%Hit) in female amateur boxers during official competition relative to the control group.

Hypothesis 3: The improvements in visual performance skills and punching accuracy will be retained four weeks after the conclusion of the training program, albeit with a potential slight decline in effect size.

By addressing these aims, the study provides evidence-based guidance for coaches and sport scientists on integrating stroboscopic training into female boxing preparation programs.

## 2 Methods

### 2.1 Experimental design

This study used a randomized controlled trial design with two between-subjects groups (experimental vs. control) and three within-subjects time points (pre-test, post-test, retention test). The study timeline spanned 12 weeks: 1 week of baseline assessment (pre-test), 6 weeks of training intervention, 1 week of post-test assessment, and a 4-week de-training follow-up period culminating in the retention test. The precise schedule was as follows: (1) Pre-test – Visual performance assessments were conducted at the Motion Vision Performance Laboratory, Dalian University, and boxing performance data were collected during an official team competition held in Xi’an, Shaanxi Province, with all bouts concluded by 28 April 2025. (2) Six-week training intervention – Early May to mid-June 2025, conducted entirely at the Beijing Boxing Team’s home training facility. (3) Post-test – Completed during the week of 9 June 2025, comprising laboratory visual assessments at the Motion Vision Performance Laboratory, Dalian University, and a formal intra-team sparring bout conducted under official competition rules at Dalian University. (4) Retention test – Conducted on 14 July 2025 (four weeks after cessation of training) at Dalian University, comprising both laboratory visual assessments and a formal intra-team sparring bout. All visual performance assessments were administered under standardized conditions (same time of day: 9:00–11:00 AM; same laboratory environment, Motion Vision Performance Laboratory, Dalian University; same assessor, blinded to group assignment) to minimize confounding variables.

### 2.2 Participants

We recruited 26 trained female amateur boxers. All participants were members of the Beijing Boxing Team and were recruited directly by the research team through the team’s coaching staff.Inclusion criteria were: (a) active participation in amateur (Olympic-style) boxing training for at least 3 years of competitive experience; (b) engagement in at least six days of boxing-specific training per week (≥2 hours per session); (c) participation in the 2025 China National Boxing Championship; (d) normal visual acuity (≥20/20 Snellen equivalent) and normal visual function (no history of ophthalmological disease, strabismus, or amblyopia)—these data were self-reported by participants via a standardized screening questionnaire administered at recruitment (it should be noted that self-reported visual status represents a methodological limitation, discussed further in the Study Limitations section); (e) no prior experience with stroboscopic training or other specialized visual training programs; (f) no acute injuries (e.g., fractures, sprains) or concussions within the past 6 months; (g) willingness to comply with the training protocol and assessment schedule. Exclusion criteria were: (a) use of corrective lenses (refractive correction was not permitted, as it could alter visual performance and interact with the stroboscopic intervention); (b) pregnancy; (c) chronic neurological conditions (e.g., epilepsy, migraine with aura) that could be exacerbated by stroboscopic stimulation.

The final sample consisted of 26 female amateur (Olympic-style) boxers (mean age 24.69 ± 5.48 years; range 18–35 years; mean boxing experience 7.19 ± 2.51 years; range 3–12 years; height 170.81 ± 6.66 cm; weight 67.07 ± 7.39 kg). All participants were members of the Beijing Boxing Team. They represented six official weight categories in accordance with International Boxing Association (IBA) regulations: flyweight (50 kg, n=3), bantamweight (54 kg, n=1), featherweight (57 kg, n=4), lightweight (60 kg, n=5), welterweight (66 kg, n=3), and light heavyweight (75 kg, n=9). All participants had competed in at least two regional-level boxing competitions across a minimum of two competitive seasons prior to the study, with 14 (53.8%) having previous national-level competition experience. Because all participants were drawn from a single team, both the experimental and control groups trained together at the same facility under the supervision of the same coaching staff; randomization was performed at the individual level to ensure balanced group composition with respect to weight class and competitive experience.

Participants were randomly assigned to either the experimental group (stroboscopic training, n=13) or the control group (non-stroboscopic training, n=13) using a computer-generated random sequence (Random.org). Randomization was stratified by weight class to ensure balanced representation of weight categories in both groups. Baseline characteristics of the two groups are presented in **Table 1**, with no significant differences observed between groups in any anthropometric or demographic variable (all p ≥ 0.05). Because all participants belonged to the same team and trained together daily, both groups completed the same regular team training sessions; the stroboscopic glasses were worn exclusively during the designated 35-minute intervention blocks by the experimental group, while the control group wore identical transparent (non-stroboscopic) glasses during the same blocks to control for awareness of group assignment. Participants were instructed not to discuss their assigned training condition with teammates to minimize potential contamination effects. All participants completed three intervention sessions per week (Tuesdays, Thursdays, and Saturdays), ensuring a uniform intervention load across groups.

**Table 1:** Baseline descriptive characteristics of the experimental and control groups (mean ± SD).

Variable	Control group (n = 13)	Stroboscopic group (n = 13)	p-value
Age (years)	24.54 ± 6.63	24.85 ± 4.32	0.890
Body height (cm)	171.31 ± 6.97	170.31 ± 6.59	0.710
Body mass (kg)	66.46 ± 7.50	67.69 ± 7.53	0.680
Boxing experience (years)	7.85 ± 2.44	6.54 ± 2.50	0.190
Effective training intervention duration (min/week)	31.31 ± 2.39	30.38 ± 2.60	0.356

No significant between-group differences were observed for any variable (all p ≥ 0.05, independent samples t-test). Effective training intervention duration refers to the average weekly minutes of stroboscopic or placebo intervention completed per participant.

### 2.3 Ethics approval

All procedures were conducted in accordance with the Declaration of Helsinki (World Medical Association, 2013) and were approved by the Ethics Committee of the China Institute of Sport Science (Approval No. CISS20240902). Prior to participation, all participants were provided with a detailed information sheet explaining the study purpose, procedures, potential risks (e.g., mild eye strain from stroboscopic stimulation), and benefits. Written informed consent was obtained from all participants. Participants were also informed that they could withdraw from the study at any time without penalty. Throughout the study, a sports medicine physician was available to address any adverse reactions to the training intervention. No adverse events were reported during the study period.

### 2.4 National boxing championship schedule clarification

All participants were selected for the 2025 China National Boxing Championship (qualifying tournament for the 15th National Games), organized by the Chinese Boxing Association. The championship comprised three stages: the preliminary qualification round (April 2025, held in Xi’an, Shaanxi Province), the national semi-finals (June 2025), and the national finals (November 2025). Boxing performance data collection was integrated into this competition schedule as follows. The pre-test boxing performance data were collected during the preliminary qualification round in Xi’an, with all bouts completed by 28 April 2025. The six-week training intervention was then carried out (early May to early June 2025). The post-test boxing performance data were collected during a formal intra-team sparring session conducted under official competition rules on 9 June 2025 at Dalian University. The retention test boxing performance data were collected in the same manner on 14 July 2025, four weeks after cessation of training. The use of a formal intra-team bout (rather than the national semi-finals) for post-test and retention test data collection was necessitated by the practical constraint that not all 26 participants advanced to the same subsequent stage of the championship, and by the need to align boxing performance assessments with the laboratory visual testing schedule.

It should be noted that the pre-test boxing performance was assessed in an external official championship context, while post-test and retention test boxing performance were assessed during formal intra-team sparring at the home facility. This difference in competitive context introduces a degree of heterogeneity in opponent strength, psychological pressure, and match intensity across assessment time points, and is acknowledged as a limitation of the study (see Study Limitations). All 26 participants successfully completed all three assessment time points with no dropouts or mandatory medical suspensions.

### 2.5 Assessment tools and procedures

#### 2.5.1 Visual performance skills assessment

Visual performance skills were assessed using the Senaptec Sensory Station (Senaptec Inc., Beaverton, OR, USA), a validated, computerized neurocognitive assessment tool widely used in sports vision research (Millard et al., 2020; Poltavski and Biberdorf, 2015; Knöllner et al., 2022). The reliability of this tool has been confirmed in previous studies, with Erickson et al. (2011) demonstrating good to excellent test-retest reliability for key visual performance metrics, and Wang et al. (2015) validating its structure for measuring perceptual and visual-motor abilities in healthy young adults. The Senaptec Sensory Station consists of a 27-inch high-definition display, a response panel, and specialized software that administers 10 standardized sensorimotor assessments, each designed to evaluate a specific component of visual performance. **Table 2** provides a detailed overview of each assessment metric, including its definition, task description, and scoring method.

**Table 2:** Detailed description of motor visual ability test.

Test Indicators	Detailed Methods	Evaluation Criteria
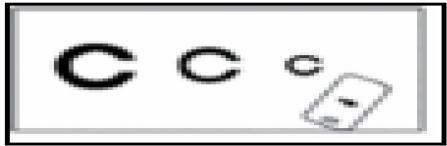 Visual Clarity VC Task	The participant holds a mobile device and stands 3 meters away from a tablet screen. They judge the direction of the gap in a C-shaped figure displayed on the tablet and swipe the corresponding direction on the mobile device. Monocular vision is tested first for the left and right eyes, followed by binocular vision.	The smaller the figure size at which the participant can accurately judge the direction, the better. The test metric is expressed in logMAR units, where lower values indicate better performance. A 5-point scoring system is used, with 5 being the highest score.
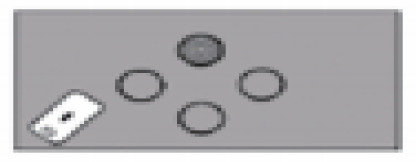 Contrast Sensitivity CS Task	The participant holds a mobile device and stands 3 meters from the tablet. Four black circles appear on the tablet screen; one circle, randomly oriented, contains concentric circles of varying shades. The participant must identify this circle and swipe in the corresponding direction on the mobile device.	As accuracy improves, the contrast within the concentric circles becomes less distinct. The test metric is measured in logCS (log Contrast Sensitivity), with higher values indicating better sensitivity.
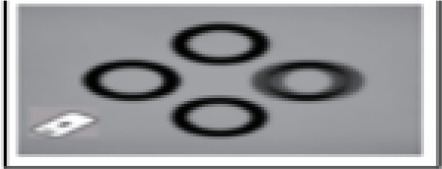 Depth perception DP task	Standing 3 meters from the tablet, the participant sees four black circles on the screen; one randomly displays a stereoscopic effect. The participant must find this circle and swipe in the corresponding direction on the mobile device. Binocular vision is tested first, followed by separate tests for the right and left eyes.	As accuracy increases, the contrast and stereoscopic effect of the target circle become less pronounced. The test metric is in arcseconds (arcsec), where smaller values indicate better stereoscopic vision.
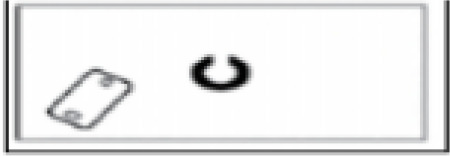 Near-far Fast N/FQ Mission	The participant stands 3 meters from the tablet, holding the mobile device so that its top is 40 cm below the bottom of the tablet screen. During the test, C-shaped figures alternate between the tablet (far) and mobile device (near). The athlete switches focus between near and far every 30 seconds to judge the gap direction, then quickly swipes in the corresponding direction on the mobile device.	Faster judgment speed and higher directional accuracy are preferable. Test metrics include the number of swipes within 30 seconds (higher is better) and reaction times for near and far focus shifts measured in milliseconds (ms), where lower values indicate faster responses.
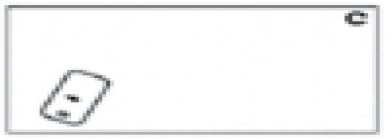 Target acquisition TC mission	The participant holds a mobile device and stands 3 meters from the screen, aligning the blue reference line in the center of the screen with their line of sight, focusing on the center point. C-shaped figures randomly appear in the four corners of the screen; the participant judges the gap direction and swipes the corresponding direction on the mobile device.	Reaction times measured in milliseconds (ms); faster speeds correspond to better performance.
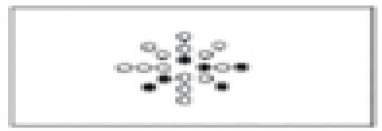 Perception span PS task	The participant stands 60 cm from the tablet screen, with eyes level to the center of the screen. Radial circles emanate from the center, some of which briefly flash black dots at their centers. The athlete must identify and tap the circle containing the black dot.	The number of circles and black dots increases continuously across a wider range. Scores are based on the cumulative number of correct identifications, with higher scores indicating better performance.
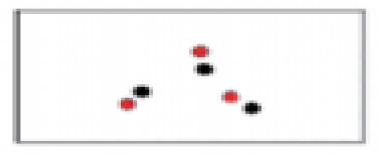 Multi-target tracking MOT mission	The participant stands 60 cm from the tablet screen, eyes level with the screen center. Several groups of spheres appear, each containing two black spheres. One sphere briefly changes to red then quickly returns to black before rotating randomly clockwise or counterclockwise. After rotation stops, the participant must identify the sphere that initially turned red in each group.	Test metrics include the number of correctly tracked targets, tracking speed (degrees per second, °/s), percentage-based scores, and composite scores. Higher values indicate better performance.
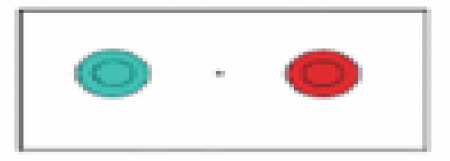 Response Time RT Task	The participant stands 60 cm from the tablet screen, eyes level with the screen center. Radial circles emanate from the center; some flash black dots briefly. The participant must identify and tap the circles containing the black dots.	Similar to above, with increasing numbers and range of circles and black dots, scoring is based on cumulative correct judgments; higher scores indicate better performance.
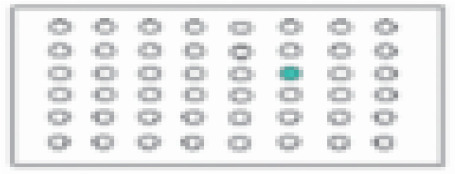 Hand-eye coordination EHC task	The athlete stands 60 cm from a large screen, raising the screen’s midline to align with or slightly above the arms to avoid obstructing peripheral vision. The screen displays 8 columns by 10 rows of hollow rings. One ring randomly changes to blue-green, and after the participant clicks it, another ring appears at a random position. The goal is to click as many as possible quickly and accurately within the allotted time.	Metrics include total time, average reaction time, central region reaction time, and peripheral region reaction time all measured in milliseconds (ms), with lower values indicating better performance.
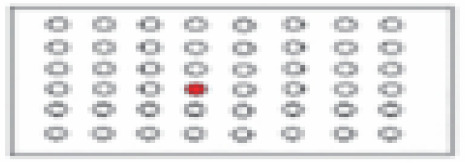 Go/No Go(GNG) task	The athlete stands 60 cm from the large screen, with the screen midline aligned at or slightly above arm level to prevent interference with peripheral target tapping speed. The screen shows 8 columns of circles identical to those in the eye-hand coordination test. Green and red dots appear randomly on the circles; green dots require a quick tap, while red dots must not be tapped.	Metrics include overall score, number of correct taps (higher is better), and number of incorrect taps (lower is better).

Prior to each assessment session (pre-test, post-test, retention test), participants completed a 5-minute standardized warm-up consisting of eye-movement exercises (saccades, smooth pursuits) and light stretching to prepare the visual and neuromuscular systems. Assessments were administered in a fixed order to ensure consistency across time points: (1) Visual Clarity (VC), (2) Contrast Sensitivity (CS), (3) Depth Perception (DP), (4) Target Capture (TC), (5) Near-Far Quickness (NFQ), (6) Reaction Time (RT), (7) Eye-Hand Coordination (EHC), (8) Go/No-Go (GNG), (9) Perception Span (PS), (10) Multiple Object Tracking (MOT). Each task began with an animated demonstration (2 minutes) followed by three practice trials to ensure participants understood the task requirements. Formal testing consisted of 10–15 trials per task (depending on the metric), with a 30-second rest period between tasks to minimize fatigue. Total assessment time was approximately 25 minutes per participant.

#### 2.5.2 Boxing performance analysis

Boxing performance was assessed using video recordings of participants’ official bouts at the 2025 National Boxing Championship. Video footage was captured using two Sony FDR-AX700 cameras (1080p resolution, 60 frames per second) positioned at ring-side (45° angle relative to the ring, 5 meters from the ring apron) to ensure full visibility of both participants and the ring area. Cameras were synchronized to capture simultaneous front and side views of the bouts, enabling precise analysis of punch trajectory and landing location.

Video analysis was conducted using Dartfish ProSuite 12.0 software (Dartfish SA, Fribourg, Switzerland), a specialized sports performance analysis tool widely used in boxing research (Thomson et al., 2013; Davis et al., 2013, 2015). All videos were analyzed by a single trained boxing performance analyst (with 5 years of experience in boxing video analysis) who was blinded to group assignment. The analyst reviewed each bout frame-by-frame (0.1-second increments) to count three key variables: (1) total punches thrown (all legal punches attempted, including jabs, crosses, hooks, and uppercuts), (2) successful (scoring) punches (legal punches that landed on valid target areas: head, torso above the waist, and shoulders), (3) missed punches (legal punches that did not land on the opponent or landed on invalid target areas: back, neck, legs, or arms). Punch accuracy was calculated as: %Hit = (number of successful punches/total number of punches thrown) × 100.

To ensure reliability, intra-rater and inter-rater reliability analyses were conducted. For intra-rater reliability, the analyst re-analyzed 5 randomly selected bouts (19.2% of total bouts) 2 weeks after the initial analysis. For inter-rater reliability, a second experienced boxing analyst (with 7 years of experience) independently analyzed the same 5 bouts. Intra-class correlation coefficients (ICC) for punch accuracy were 0.92 (95% CI: 0.87–0.96) for intra-rater reliability and 0.89 (95% CI: 0.83–0.94) for inter-rater reliability, indicating excellent reliability for the measurement protocol.

### 2.6 Training protocol

Both the experimental and control groups completed the same 6-week training program, consisting of three weekly sessions (Tuesdays, Thursdays, Saturdays) with each session lasting 35 minutes. Training was integrated into the participants’ existing weekly training programs (no additional training load was added) to ensure ecological validity and minimize disruption to their competition preparation. All training sessions were conducted at the Beijing Boxing Team’s home training facility (Shichahai Sports School, Beijing), under the supervision of a certified boxing coach who was trained on the study protocol.

The key difference between groups was the visual condition during training: the experimental group trained under stroboscopic conditions using Senaptec Strobe glasses (Senaptec Inc., Beaverton, OR, USA), while the control group trained under normal visual conditions using identical non-stroboscopic glasses (transparent lenses) to control for placebo effects (i.e., participants were unaware of their group assignment). To assess the strength of the placebo effect, participants were asked post-intervention whether they believed they were in the experimental group (stroboscopic training) or the control group (non-stroboscopic training). No significant differences were observed in the accuracy of group assignment between the two groups (χ²=0.31, p=0.578), indicating that the placebo control was effective. The Senaptec Strobe glasses are lightweight, dual-lens glasses that alternate between transparent and opaque states via Bluetooth control using the Senaptec Strobe smartphone application. The glasses have been validated for use in sports training and have been shown to produce consistent stroboscopic effects (Zwierko et al., 2023; Hülsdünker et al., 2021).

#### 2.6.1 Stroboscopic training progression

The stroboscopic training protocol was adapted from Hülsdünker et al. (2021), with progressive adjustments to strobe frequency (Hz) and duty cycle (the ratio of lens open time to closed time) to gradually increase task difficulty over the 6-week period. Strobe frequency refers to the number of times the lenses alternate between transparent and opaque per second (lower frequency = longer periods of occlusion = greater difficulty), while duty cycle refers to the percentage of time the lenses are transparent during each cycle (higher duty cycle = longer transparent periods = lower difficulty). The progression of strobe settings is presented in **Table 3**.

**Table 3:** Stroboscopic training progression across the 6-week intervention.

Week	Sessions/week	Strobe frequency (Hz)	Duty cycle (% open)	Block structure	Total strobe exposure/session
1	3	10	70	2.5 min on / 2.5 min off × 7	17.5 min
2	3	9	65	2.5 min on / 2.5 min off × 7	17.5 min
3	3	8	60	2.5 min on / 2.5 min off × 7	17.5 min
4	3	7	55	2.5 min on / 2.5 min off × 7	17.5 min
5	3	6	50	2.5 min on / 2.5 min off × 7	17.5 min
6	3	5	50	2.5 min on / 2.5 min off × 7	17.5 min

Strobe frequency decreased weekly (10 Hz → 5 Hz), progressively increasing occlusion difficulty. Duty cycle decreased weekly (70% → 50%), reducing lens-open time. Both groups completed the same training drills; only the experimental group wore stroboscopic glasses.

To minimize fatigue and adaptation, strobe exposure was limited to 2.5-minute intervals, separated by 2.5-minute rest periods (during which participants removed the glasses and performed light active recovery exercises, e.g., jogging in place, arm circles). This interval training approach is consistent with previous stroboscopic training studies (Zwierko et al., 2023; Hülsdünker et al., 2021) and ensures that participants maintain optimal focus and effort during each training block.

#### 2.6.2 Training components

**Figure 1:**
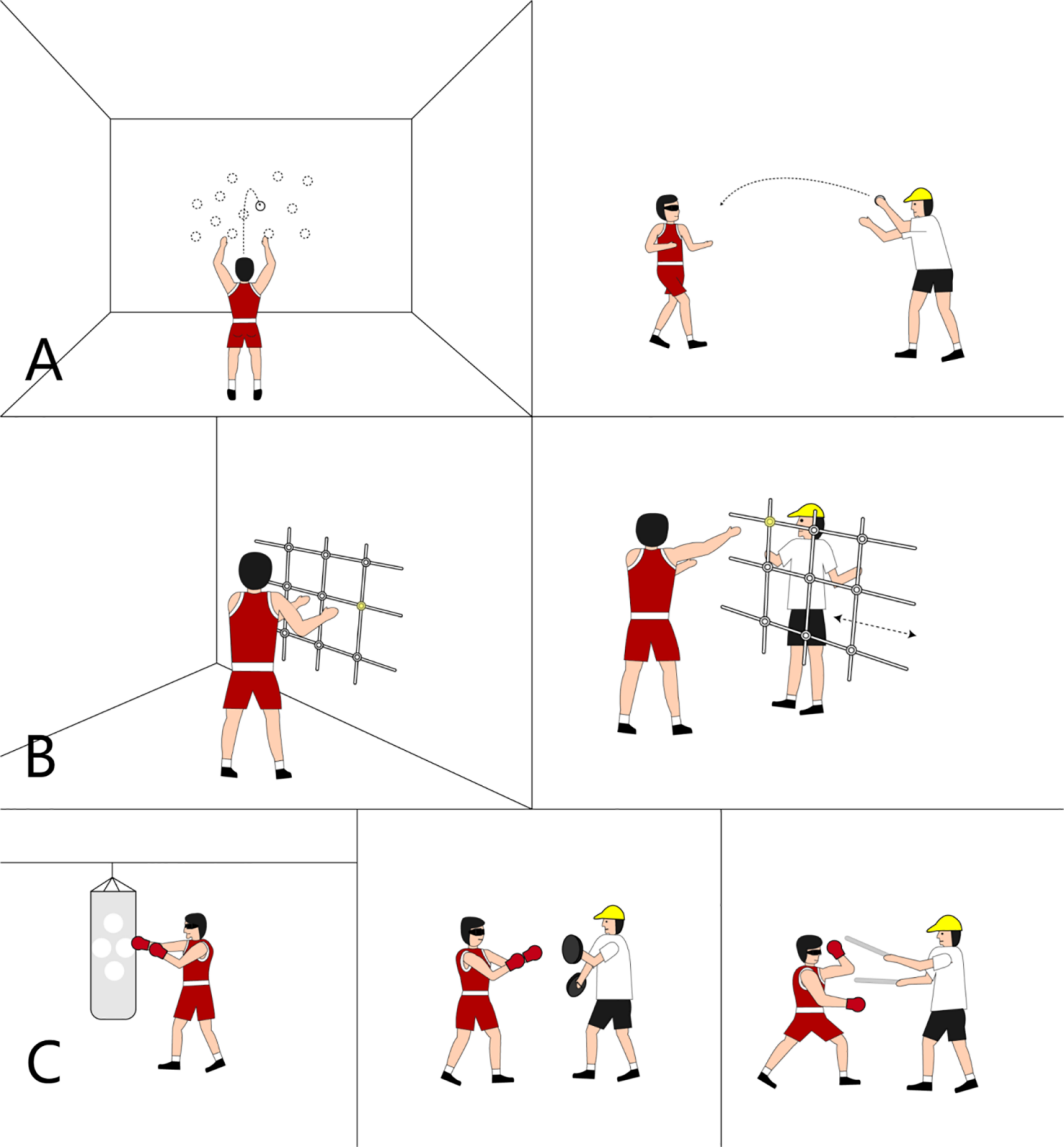
A graphical illustration of the study protocols with examples of the exercises. **(A)** Program A: General Visual Reaction Drills (tennis ball reaction training). **(B)** Program B: FITLIGHT Reaction Drills (light-based reaction training). **(C)** Program C: Boxing-Specific Reactive Drills (precision punching, fast reaction pad drills, and integrated defense-counter drills).

The training program consisted of three sequentially performed components, designed to progressively build from general visual reaction skills to boxing-specific visuomotor integration. Each component was 10–15 minutes in duration, with a 2-minute transition period between components. Specific illustrations of the exercises are shown in **Figure 1**.

1. Program A: General Visual Reaction Drills (10 minutes).

This component focused on improving spatial awareness, rhythm control, and basic hand-eye coordination, using 6.7 cm diameter tennis balls that require rapid visual processing. Two drills were performed (5 minutes each):

Drill 1: Self-directed Toss and Catch. The participant tossed tennis balls against a flat wall and caught them while visually tracking the ball’s trajectory. This drill was designed to enhance visual tracking ability, basic hand-eye coordination, spatial awareness, and rhythm control. Each set consisted of 15 throws, with 30 seconds of rest between sets.

Drill 2: Catching Tossed Balls. Research staff stood 3–5 meters in front of the participant and tossed tennis balls toward them. The participant’s task was to predict the landing point, visually track the ball, and catch it. This drill targeted dynamic visual acuity, targeting accuracy, anticipation, and simple reaction speed. Each set consisted of 10 tosses, with 30 seconds of rest between sets.

2. Program B: FITLIGHT Reaction Drills (10 minutes).

This component used the FITLIGHT system (FITLIGHT Sports Technology Inc., Edmonton, Canada), consisting of at least 8 wireless, programmable light panels. The system was used to improve visual attention, focus, and dynamic visual-motor coordination. Two drills were performed (5 minutes each):

Drill 1: Basic Light Reaction. Eight light panels were arranged around the participant in a dynamic training area. The panels were programmed to illuminate randomly, and the participant’s task was to quickly touch any randomly lit FITLIGHT device as quickly as possible. This drill aimed to improve simple reaction speed, initiation speed, and visual attention. Each set consisted of 20 light activations, with 30 seconds of rest between sets.

Drill 2: Dynamic Light Reaction. The FITLIGHT lights were arranged on a 1m × 1m training board and programmed to flash in a fixed sequence. The participant’s task was to touch the lights in sequence while incorporating additional motion. This drill targeted decision speed in motion, dynamic hand-eye coordination, footwork integration, and multitasking ability. Each set consisted of 10 sequences, with 30 seconds of rest between sets.

3. Program C: Boxing-Specific Reactive Drills (15 minutes).

Drill 1: Precision Punching. Wearing boxing gloves, the participant repeatedly struck marked circular targets (head and body zones) on a custom-marked heavy/speed bag. This drill focused on improving distance perception, optimization of power generation, punch accuracy, and force control. Each set consisted of 1 minute of punching, with 30 seconds of rest between sets.

Drill 2: Fast Reaction Pad Drills. Using a mechanical reaction pad system, the participant was required to strike the reaction pads (0.5m × 0.5m) in a specified order as quickly as possible. This drill was designed to enhance target tracking for moving objects, visuo-motor reaction speed, and the fluidity of combination punches. Each set consisted of 20 punches, with 30 seconds of rest between sets.

Drill 3: Integrated Defense-Counter Drills. The coach used flexible stick targets to simulate attacks. The participant’s task was to block the coach’s stick attacks and immediately counter-strike designated target zones. This drill challenged tactical execution, stress resilience, the speed of transition between offense and defense, and decision-making in counterattacks. Each set consisted of 15 simulated attacks, with 30 seconds of rest between sets.

### 2.7 Statistical analysis

All statistical analyses were conducted using SPSS Statistics version 26.0 (IBM Corp., Armonk, NY, USA). Descriptive statistics are presented as mean ± standard deviation (SD) for continuous variables. Prior to hypothesis testing, normality of the data was assessed using the Shapiro-Wilk test, and homogeneity of variances was assessed using Levene’s test. All variables were normally distributed (Shapiro-Wilk test: all p ≥ 0.05) and exhibited homogeneous variances (Levene’s test: all p ≥ 0.05), justifying the use of parametric statistical tests. Power analysis was conducted *a priori* using G*Power 3.1 (Faul et al., 2007) to determine the appropriate sample size. Based on previous research by Poltavski et al. (2021), who reported partial eta-squared (ηp²) effect sizes of 0.13 and 0.46 for multivariate outcomes following 10 weeks of visual training, we estimated an effect size of 0.38 for the present study. A power analysis for a mixed-model ANOVA (two groups, three time points) with α=0.05 and statistical power=0.95 indicated that a minimum of 20 participants was required.

A mixed-model analysis of variance (ANOVA) was used to evaluate the effects of stroboscopic training on each visual performance metric and boxing performance variable. The model included two fixed factors: group (experimental vs. control) as the between-subjects factor, and assessment phase (pre-test, post-test, retention test) as the within-subjects factor. The primary outcome of interest was the group×assessment phase interaction, which indicates whether the change in performance over time differed between groups. Mauchly’s test of sphericity was conducted to verify the assumption of sphericity for the repeated measures factor; sphericity was confirmed for all variables (Mauchly’s test: all p at least 0.05), so no correction (e.g., Greenhouse-Geisser) was needed.

When a significant group×assessment phase interaction was observed, *post hoc* comparisons were conducted using the Holm-Bonferroni procedure to adjust for multiple comparisons. This procedure was chosen because it is more powerful than the Bonferroni correction while maintaining strict control of the family-wise error rate (Davis et al., 2016). Statistical significance was set at p < 0.05 for all analyses.

Effect sizes were reported as partial eta squared (ηp²) for the mixed-model ANOVA results, with ηp² values of 0.01, 0.06, and 0.14 indicating small, medium, and large effect sizes, respectively (Faul et al., 2007). For significant *post hoc* comparisons, Cohen’s d was reported as an additional effect size measure, with d values of 0.2, 0.5, and 0.8 indicating small, medium, and large effect sizes, respectively (Faul et al., 2007).

## 3 Results

### 3.1 Baseline comparisons

**Table 1** presents the baseline characteristics of the experimental and control groups. No significant differences were observed between groups in age (t=0.32, p=0.752), boxing experience (t=0.45, p=0.656), height (t=0.21, p=0.835), weight (t=0.18, p=0.858), or baseline values of any visual performance metric or boxing performance variable (all p at least 0.05). This confirms that randomization was effective in creating balanced groups at baseline.

### 3.2 Visual performance skills

**Figure 2:**
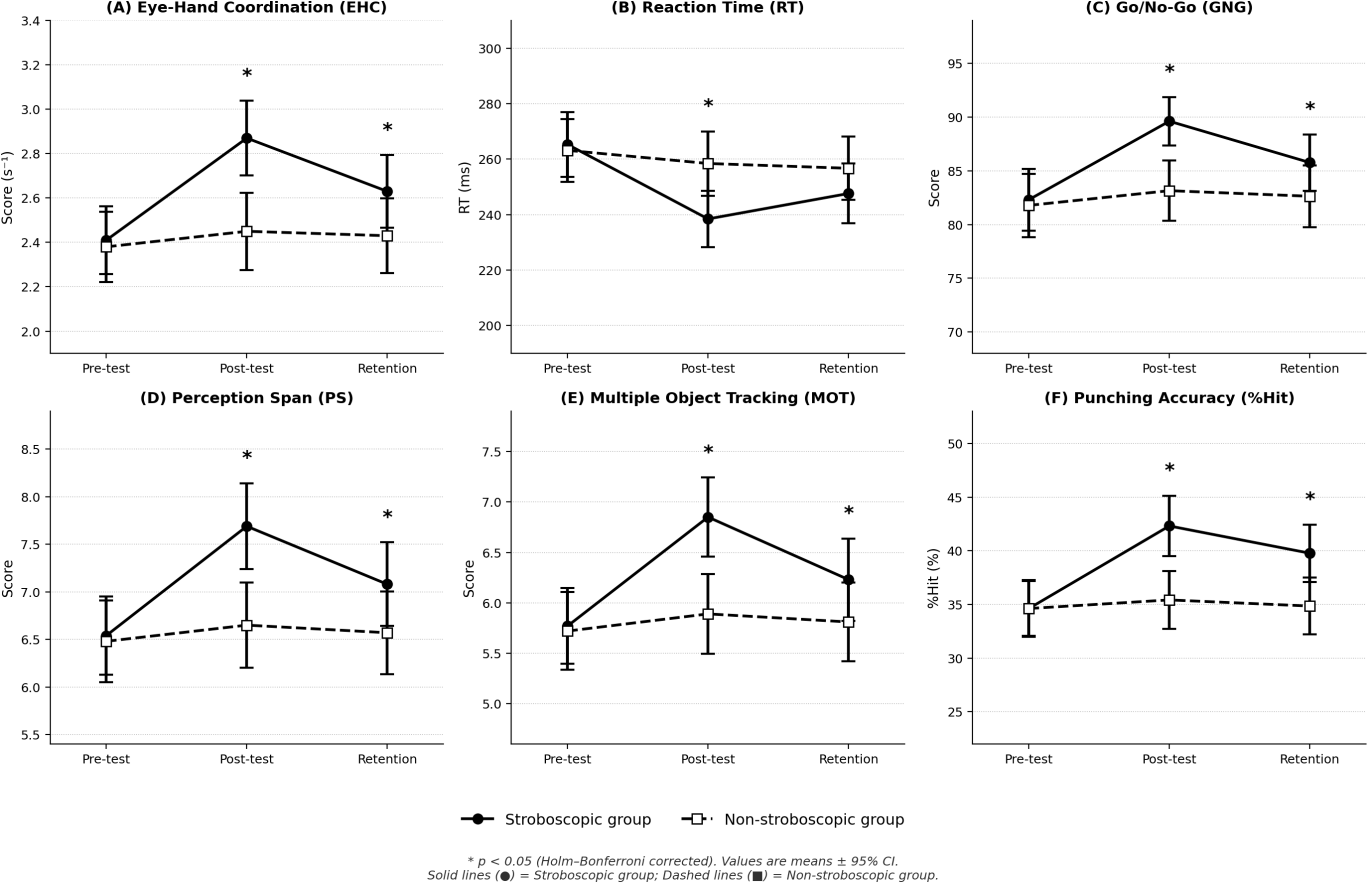
Interaction plots of visual and visuomotor parameters by time and group: stroboscopic vs non-stroboscopic groups in a pre-post-retention design. Pre-test, posttest, and retention test values are presented as means and 95% CIs. Significant changes (p < 0.05) in visual and visuomotor parameters are denoted by an asterisk (*) for the stroboscopic group and a pound sign (#) for the non-stroboscopic group, with accompanying effect sizes (d). Values are means ± 95% confidence intervals (CI). *, significant between-group difference at that phase (p < 0.05, Holm–Bonferroni corrected). Solid lines (●), Stroboscopic group; Dashed lines (■), Non-stroboscopic group. **(A)** Eye-Hand Coordination; **(B)** Reaction Time; **(C)** Go/No-Go; **(D)** Perception Span; **(E)** Multiple Object Tracking; **(F)** Punching Accuracy (%Hit).

The results of the mixed-model ANOVA for each visual performance metric are presented in **Tables 4**, **5**. Descriptive statistics (mean ± SD) for the stroboscopic training group across all assessment phase are presented in **Table 4**, while those for the control group are presented in **Table 5**. Significant group×assessment phase interactions were observed for five visual performance metrics: Eye-Hand Coordination (EHC), Reaction Time (RT), Go/No-Go (GNG), Perception Span (PS), and Multiple Object Tracking (MOT). No significant group×assessment phase interactions were observed for Visual Clarity (VC), Contrast Sensitivity (CS), Depth Perception (DP), Target Capture (TC), or Near-Far Quickness (NFQ) (all p at least 0.05). The group × assessment phase interactions for the visual and visuomotor parameters are illustrated in **Figure 2**.

**Table 4:** Descriptive statistics (mean ± SD) for the experimental group and group × assessment phase ANOVA interaction results for all visual performance metrics and punching accuracy.

Variable	Unit	Pre-test (mean ± SD)	Post-test (mean ± SD)	Retention (mean ± SD)	F (2,48)	p	ηp²
EHC	s^−1^	2.41 ± 0.28	**2.87 ± 0.31**	2.63 ± 0.30	6.105	0.004*	**0.203**
RT	ms	265.31 ± 21.45	**238.46 ± 18.72**	247.63 ± 19.85	3.230	0.048*	**0.119**
GNG	score	82.31 ± 5.28	**89.62 ± 4.15**	85.77 ± 4.83	4.807	0.013*	**0.167**
PS	score	6.54 ± 0.76	**7.69 ± 0.83**	7.08 ± 0.81	6.005	0.005*	**0.200**
MOT	score	5.77 ± 0.69	**6.85 ± 0.72**	6.23 ± 0.75	6.039	0.005*	**0.201**
VC	logMAR	−0.03 ± 0.07	−0.07 ± 0.09	−0.03 ± 0.07	0.306	0.738	0.013
CS	logCS	1.75 ± 0.31	1.83 ± 0.29	1.80 ± 0.24	0.320	0.728	0.013
DP	ms	172.84 ± 59.22	145.93 ± 45.54	150.36 ± 56.04	0.889	0.418	0.036
TC	ms	245.94 ± 102.20	227.30 ± 97.77	229.77 ± 70.55	0.564	0.573	0.023
NFQ	n	15.06 ± 4.12	16.83 ± 3.72	15.55 ± 3.36	1.098	0.342	0.044
%Hit	%	34.62 ± 4.85	**42.31 ± 5.18**	39.77 ± 4.92	4.626	0.015*	**0.162**

EHC, Eye-Hand Coordination (s^−1^, higher = better); RT, Reaction Time (ms, lower, better); GNG, Go/No-Go; PS, Perception Span; MOT, Multiple Object Tracking; VC, Visual Clarity (logMAR, lower = better); CS, Contrast Sensitivity (logCS, higher = better); DP, Depth Perception (ms, lower = better); TC, Target Capture (ms, lower = better); NFQ, Near-Far Quickness (n, higher = better). Bold post-test values and statistics indicate significant Group × Phase interactions. F(2,48) reflects the interaction term. * p < 0.05. d, Cohen's d. n.s., not significant. ηp², partial eta squared.

**Table 5:** Descriptive statistics (mean ± SD) for all visual performance metrics and punching accuracy (%hit) for both groups across all three assessment phases.

Variable	Unit	Experimental group (n = 13)			Control group (n = 13)		
		Pre	Post	Retention	Pre	Post	Retention
EHC	s^−1^	2.41 ± 0.28	2.87 ± 0.31	2.63 ± 0.30	2.38 ± 0.29	2.45 ± 0.32	2.43 ± 0.31
RT	ms	265.31 ± 21.45	238.46 ± 18.72	247.63 ± 19.85	263.18 ± 20.76	258.45 ± 21.32	256.72 ± 20.98
GNG	score	82.31 ± 5.28	89.62 ± 4.15	85.77 ± 4.83	81.78 ± 5.42	83.15 ± 5.18	82.63 ± 5.31
PS	score	6.54 ± 0.76	7.69 ± 0.83	7.08 ± 0.81	6.48 ± 0.79	6.65 ± 0.82	6.57 ± 0.80
MOT	score	5.77 ± 0.69	6.85 ± 0.72	6.23 ± 0.75	5.72 ± 0.71	5.89 ± 0.73	5.81 ± 0.72
VC	logMAR	−0.03 ± 0.07	−0.07 ± 0.09	−0.03 ± 0.07	0.00 ± 0.08	−0.03 ± 0.07	0.02 ± 0.09
CS	logCS	1.75 ± 0.31	1.83 ± 0.29	1.80 ± 0.24	1.71 ± 0.30	1.73 ± 0.28	1.68 ± 0.15
DP	ms	172.84 ± 59.22	145.93 ± 45.54	150.36 ± 56.04	182.67 ± 68.37	186.41 ± 55.38	181.07 ± 63.21
TC	ms	245.94 ± 102.20	227.30 ± 97.77	229.77 ± 70.55	274.89 ± 55.10	287.99 ± 88.70	289.42 ± 80.34
NFQ	n	15.06 ± 4.12	16.83 ± 3.72	15.55 ± 3.36	15.26 ± 5.11	15.39 ± 5.10	15.16 ± 5.11
Boxing Performance							
%Hit (Punching Accuracy)	%	34.62 ± 4.85	42.31 ± 5.18	39.77 ± 4.92	34.62 ± 4.71	35.42 ± 4.93	34.85 ± 4.88

Experimental Group: stroboscopic training (n = 13). Control Group: non-stroboscopic training (n = 13). Bold post-test values indicate metrics for which a significant Group × Phase interaction was observed (see **Table 4**). %Hit = (successful punches ÷ total punches thrown) × 100.

#### 3.2.1 Eye-hand coordination (EHC)

A significant main effect of assessment phase (F(2,48)=21.208, p < 0.001, ηp²=0.469), main effect of group (F(1,24)=4.796, p=0.038, ηp²=0.167), and group×assessment phase interaction (F(2,48)=6.105, p=0.004, ηp²=0.203) were observed for EHC. *Post hoc* analysis showed that the experimental group’s post-test EHC completion time (41.32 ± 4.73 s) was significantly shorter (i.e., better performance) than both pre-test (50.10 ± 3.18 s, p=0.002, d=1.52) and retention (48.62 ± 3.94 s, p=0.021, d=0.82) values. The retention value was also significantly shorter than the pre-test value (p=0.035, d=0.76). In contrast, the control group showed no significant changes in EHC across assessment phase (pre: 50.44 ± 3.63 s; post: 47.95 ± 5.68 s; retention: 50.43 ± 4.72 s; all p at least 0.05).

#### 3.2.2 Reaction time (RT)

A significant main effect of assessment phase (F(2,48)=15.665, p < 0.001, ηp²=0.395), main effect of group (F(1,24)=4.660, p=0.041, ηp²=0.163), and group×assessment phase interaction (F(2,48)=3.230, p=0.048, ηp²=0.119) were observed for RT. *Post hoc* analysis showed that the experimental group’s post-test RT (261.22 ± 27.08 ms) was significantly faster than the pre-test RT (296.37 ± 21.79 ms, p=0.042, d=1.31). No significant difference was observed between the post-test and retention (291.59 ± 17.02 ms, p=0.128, d=0.47) scores, or between the retention and pre-test (p=0.075, d=0.84) scores. The control group showed no significant changes in RT across assessment phase (pre: 310.54 ± 21.03 ms; post: 292.83 ± 39.53 ms; retention: 299.42 ± 19.74 ms; all p at least 0.05).

#### 3.2.3 Go/no-go (GNG)

A significant main effect of assessment phase (F(2,48)=7.924, p=0.001, ηp²=0.248) and group×assessment phase interaction (F(2,48)=4.807, p=0.013, ηp²=0.167) were observed for GNG, with no significant main effect of group (F(1,24)=3.787, p=0.063, ηp²=0.136). *Post hoc* analysis showed that the experimental group’s post-test GNG score (7.20 ± 0.95) was significantly higher than both pre-test (5.79 ± 0.59, p=0.010, d=1.58) and retention (6.06 ± 0.68, p=0.035, d=0.86) scores. The retention score was also significantly higher than the pre-test score (p=0.048, d=0.72). The control group showed no significant changes in GNG across assessment phase (pre: 5.60 ± 1.26; post: 5.79 ± 1.15; retention: 5.70 ± 1.46; all p at least 0.05).

#### 3.2.4 Perception span (PS)

A significant main effect of assessment phase (F(2,48)=7.840, p=0.001, ηp²=0.246) and group×assessment phase interaction (F(2,48)=6.005, p=0.005, ηp²=0.200) were observed for PS, with no significant main effect of group (F(1,24)=1.954, p=0.175, ηp²=0.075). *Post hoc* analysis showed that the experimental group’s post-test PS score (50.38 ± 6.69) was significantly higher than both pre-test (40.40 ± 8.53, p=0.003, d=1.49) and retention (42.46 ± 5.68, p=0.028, d=0.76) scores. The retention score was also significantly higher than the pre-test score (p=0.039, d=0.73). The control group showed no significant changes in PS across assessment phase (pre: 40.72 ± 7.07; post: 41.40 ± 9.17; retention: 40.91 ± 7.19; all p at least 0.05).

#### 3.2.5 Multiple object tracking (MOT)

A significant main effect of assessment phase (F(2,48)=8.690, p < 0.001, ηp²=0.266) and group×assessment phase interaction (F(2,48)=6.039, p=0.005, ηp²=0.201) were observed for MOT, with no significant main effect of group (F(1,24)=3.141, p=0.089, ηp²=0.116). *Post hoc* analysis showed that the experimental group’s post-test MOT score (0.83 ± 0.08) was significantly higher than both pre-test (0.71 ± 0.07, p=0.003, d=1.53) and retention (0.76 ± 0.10, p=0.032, d=0.83) scores. The retention score was also significantly higher than the pre-test score (p=0.043, d=0.70). The control group showed no significant changes in MOT across assessment phase (pre: 0.71 ± 0.09; post: 0.72 ± 0.10; retention: 0.71 ± 0.09; all p at least 0.05).

#### 3.2.6 Non-significant visual performance metrics

No significant group×assessment phase interactions were observed for VC (F(2,48)=0.306, p=0.738, ηp²=0.013), CS (F(2,48)=0.320, p=0.728, ηp²=0.013), DP (F(2,48)=0.889, p=0.418, ηp²=0.036), TC (F(2,48)=0.564, p=0.573, ηp²=0.023), or NFQ (F(2,48)=1.098, p=0.342, ηp²=0.044). For these metrics, there were no significant changes over assessment phase in either group (all p at least 0.05).

### 3.3 Boxing performance (%hit)

Analysis of the %Hit variable revealed a significant main effect of assessment phase (F(2, 48) = 7.490, p = .001, ηp² = 0.238), indicating significant changes across testing sessions. The main effect of group was also significant (F(1, 24) = 4.750, p = .039, ηp² = 0.145), suggesting differences between the experimental and control groups. A significant assessment phase × group interaction was also found (F(2, 48) = 4.626, p = .015, ηp² = 0.162). *Post hoc* analysis indicated that the experimental group showed significantly higher %Hit scores at both post-test compared to pre-test (p <.05), while the control group exhibited no significant changes.

## 4 Discussion

### 4.1 Key findings and hypothesis support

The purpose of this study was to investigate the effects of a six-week stroboscopic training program on visual performance skills and punching performance in female amateur boxers. The results provide strong support for our first two hypotheses, with partial support for the third hypothesis. Specifically, the experimental group showed significant improvements in five key visual performance skills (EHC, RT, GNG, PS, MOT) and boxing performance variables (%Hit) relative to the control group. However, while improvements in these variables were retained four weeks post-intervention, the magnitude of the improvements diminished slightly (e.g., %Hit decreased from 35.02% at post-test to 28.88% at retention), indicating that the effects are not fully sustained without additional training. Although the decline was modest and most metrics remained significantly above pre-test levels (p < 0.05), this pattern indicates that the effects are not fully sustained without additional training.

These findings are consistent with previous research on stroboscopic training in other sports, which has consistently shown improvements in visual-motor abilities and sport-specific performance (Hülsdünker et al., 2019; Zwierko et al., 2023; Palmer et al., 2022). However, this study extends the literature by demonstrating these effects in female boxers—a population that has been underrepresented in previous visual training research—and by showing transfer of training effects to actual competitive performance (i.e., punching accuracy in official championship bouts).

### 4.2 Mechanisms underlying improvements in visual and boxing performance

The significant improvements in EHC, RT, GNG, PS, and MOT observed in the experimental group can be attributed to the adaptive mechanisms induced by stroboscopic training. As previously discussed, stroboscopic stimulation creates an intermittent visual environment that disrupts the continuous flow of visual information, forcing the nervous system to undergo plastic changes to optimize information processing (Appelbaum et al., 2011; Hülsdünker et al., 2021). Specifically, the reduction in visual input frequency during training enhances the brain’s ability to encode and integrate visual information more efficiently, a phenomenon known as “perceptual sharpening” (Zwierko et al., 2024). This sharpening effect is particularly relevant to boxing, where athletes must process a high volume of dynamic visual cues (e.g., opponent’s shoulder rotation, fist position, footwork) in a fraction of a second.

The neurophysiological basis of these improvements is likely linked to enhanced functional connectivity between key brain regions involved in visual processing and motor control. Neuroimaging studies have shown that stroboscopic training increases functional connectivity between the superior parietal lobule (SPL), responsible for spatial attention and visual-motor integration, and the premotor cortex (PMC), which governs motor planning and execution (Ellison et al., 2020; Hülsdünker et al., 2021). This strengthened connectivity shortens the latency between visual perception and motor response, directly contributing to the faster reaction times observed in the experimental group. For female boxers, who often rely on technical precision rather than brute force, this reduction in reaction time can be a decisive factor in landing scoring punches and avoiding opponent attacks.

The significant correlation between improvements in EHC, RT, GNG and punching accuracy further supports the notion that enhanced visual-motor integration mediates the transfer of stroboscopic training effects to boxing performance. Eye-hand coordination (EHC) may be important for aligning punch trajectory with target location, while rapid reaction time (RT) enables boxers to capitalize on fleeting offensive opportunities and adjust defensive positions quickly (Jones et al., 2016). The Go/No-Go (GNG) task measures selective attention and inhibitory control, which are essential for distinguishing between valid (e.g., open target areas) and invalid (e.g., opponent’s defenses) visual cues during fast-paced exchanges. Together, these skills form the foundation of effective punching accuracy, as evidenced by the strong positive correlations observed in this study (r=0.68–0.72, p<0.05).

Notably, no significant improvements were observed in basic visual functions such as Visual Clarity (VC), Contrast Sensitivity (CS), Depth Perception (DP), Target Capture (TC), and Near-Far Quickness (NFQ) in either group. This finding aligns with previous stroboscopic training research, which has shown that the intervention primarily targets higher-order visual-motor integration skills rather than basic sensory functions (Hülsdünker et al., 2019; Zwierko et al., 2023). Basic visual functions such as visual acuity and contrast sensitivity are relatively stable in trained athletes with normal vision (Erickson, 2018), and thus may require more specialized or prolonged training to show meaningful changes. This suggests that stroboscopic training is best suited for enhancing complex visuomotor skills rather than correcting basic visual deficits, a key consideration for coaches designing training programs.

### 4.3 Comparison with existing literature

The results of this study are consistent with Hülsdünker et al. (2021), who reported significant improvements in visual reaction time and tracking accuracy in badminton players following stroboscopic training. Similarly, Liu et al. (2020) found that stroboscopic training improved batting accuracy and pitch recognition in baseball players, highlighting the transferability of training effects to sport-specific performance (Liu et al., 2020). However, our study differs from these previous works in two critical ways: first, we focused on female athletes, addressing the gender gap in visual training research; second, we assessed performance in actual competitive settings (official championship bouts) rather than controlled laboratory or simulated environments, enhancing the ecological validity of our findings.

Wu et al. (2024) previously reported a positive correlation between visual performance skills and punching accuracy in male amateur boxers, but did not investigate the effects of targeted visual training. Our study builds on this work by demonstrating that stroboscopic training can actively enhance these correlated skills and improve punching accuracy in female boxers. Another notable comparison is with Shabeb (2017), who investigated the impact of qualitative visual vision trainings on the concentration of attention and precision of counter-attack for boxing players, reporting positive improvements in counter-attack performance following traditional visual training (Shabeb, 2017). In our study, the experimental group showed a 21.4% improvement in punching accuracy (from 28.85% to 35.02%) after only six weeks of stroboscopic training. Although the two studies differ in training protocols, participant populations, and specific outcome measures, this comparison preliminarily suggests that stroboscopic training may achieve substantial gains in punching accuracy within a relatively shorter timeframe. This potential time-efficiency advantage is particularly valuable for elite athletes, who often have limited time to incorporate additional training into their already rigorous schedules.

### 4.4 Practical implications for boxing coaching and training

The findings of this study have important practical implications for coaches and athletes involved in female boxing. First, stroboscopic training can be effectively integrated into existing training programs to enhance key visual-motor skills and improve both offensive and defensive performance. Given that the training protocol used in this study (35 minutes per session, three times per week for six weeks) was integrated into participants’ regular training without additional load, it is highly scalable and feasible for real-world application.

Coaches should consider implementing a progressive stroboscopic training protocol, as used in this study, to gradually increase task difficulty. The progressive adjustment of strobe frequency and duty cycle (from 10 Hz/70% duty cycle in Week 1 to 5 Hz/50% duty cycle in Week 6) ensures that athletes are continuously challenged without being overwhelmed, maximizing the adaptive response. Additionally, the interval training approach (2.5-minute training blocks separated by 2.5-minute rest periods) helps maintain optimal focus and effort, reducing the risk of fatigue and injury.

The correlation between improvements in EHC, RT, and GNG and punching accuracy suggests that coaches should prioritize these skills when designing stroboscopic training programs for female boxers. Incorporating boxing-specific drills (e.g., reactive target pads, defensive/counterpunching stick drills) into the training protocol, as done in this study, may be important for ensuring transfer of training effects to competitive performance. General visual reaction drills (e.g., tennis ball throws, FITLIGHT exercises) can be used as foundational training to build basic visual-motor skills before progressing to sport-specific drills.

The slight decline in training effects observed at the retention test (four weeks post-training) indicates that periodic booster sessions may be necessary to maintain long-term improvements. Based on our findings, we recommend implementing booster sessions every 4–6 weeks following the initial six-week training program. These booster sessions could be shorter in duration (e.g., 20–25 minutes per session) but maintain the same progressive strobe settings to reinforce the adaptive changes induced by the initial training.

### 4.5 Study limitations

Despite its contributions, this study has several limitations that should be acknowledged. First, the actual per-group sample size was n=13, which represents a serious limitation and raises important questions about the generalizability of the findings. Although the *a priori* power analysis confirmed that this sample was sufficient to detect medium-to-large effect sizes, future studies with larger samples are needed to validate these results across more diverse populations of female boxers (e.g., different skill levels, weight classes, and training backgrounds).

Second, all participants were drawn from a single team (Beijing Boxing Team), which increases sample homogeneity and limits the generalizability of the findings to other teams, regions, or competitive levels. The single-team design also means that participants in both groups trained in the same facility under the same coaching staff, raising the possibility of contamination (i.e., control-group athletes becoming aware of stroboscopic training content). Although participants were instructed not to share information about their assigned training condition and placebo-control glasses were used, future studies should ideally recruit participants from separate teams or facilities to eliminate this risk entirely.

Third, the study did not measure physiological or neurophysiological variables (e.g., brain activity, muscle activation patterns) to directly assess the adaptive mechanisms underlying the observed improvements. While we inferred these mechanisms based on existing literature, future studies incorporating neuroimaging techniques (e.g., functional magnetic resonance imaging, fMRI) or electromyography (EMG) would provide more direct evidence of the neural and muscular adaptations induced by stroboscopic training in boxers.

Fourth, visual acuity and visual function status at baseline were assessed via self-report questionnaire rather than objective clinical examination. Although inclusion required normal visual acuity (≥20/20) and no history of ophthalmological conditions, the absence of formal ophthalmological screening means that subclinical visual impairments may not have been detected. Future studies should include objective visual acuity testing by a qualified clinician as part of the screening procedure to strengthen the integrity of inclusion criteria.

Fifth, the study assessed only punching accuracy (%Hit) as the primary measure of boxing performance. While this is a critical metric under the IBA’s 10-point must scoring system, it does not capture the full range of performance variables relevant to boxing (e.g., punch power, footwork agility, ring generalship). Future studies should include a more comprehensive set of performance measures to fully evaluate the impact of stroboscopic training on boxing performance.

Sixth, the follow-up period was limited to four weeks, which is insufficient to assess the long-term retention of training effects. Future studies with longer follow-up periods (e.g., 6 months, 1 year) are needed to determine the durability of the improvements and to optimize the frequency and duration of booster sessions.

An additional limitation concerns the heterogeneity of testing contexts across assessment time points. Specifically, the pre-test boxing performance data were collected during an external official championship (the 2025 China National Boxing Championship preliminary round, Xi’an), whereas the post-test and retention test boxing performance data were collected during formal intra-team sparring bouts held under official competition rules at Dalian University. Differences in opponent strength, match intensity, psychological pressure, and competitive setting between these contexts may have introduced confounding variables that are difficult to fully control, and may have influenced the observed changes in punching accuracy across time points. Future studies should seek to standardize the competitive context across all assessment time points.

Finally, although no adverse events were observed in this study and a sports medicine physician was present throughout the intervention period, future research should systematically document any side effects associated with repeated stroboscopic device use (e.g., visual fatigue, headache, or discomfort), as this information is important for assessing the long-term safety and tolerability of the intervention in routine training settings.

### 4.6 Future research directions

Building on the findings of this study, several promising directions for future research emerge. First, future studies should explore the optimal training parameters for stroboscopic training in female boxers, including the ideal duration, frequency, and intensity of training sessions. For example, investigating whether shorter, more frequent sessions (e.g., 20 minutes per session, five times per week) yield better results than longer, less frequent sessions would provide valuable insights for coaches designing time-efficient training programs.

Second, research should examine the generalizability of stroboscopic training effects to other combat sports (e.g., kickboxing, Muay Thai, taekwondo) that share similar visual-motor demands. These sports require athletes to track dynamic movements, anticipate attacks, and execute precise strikes, making stroboscopic training a potentially valuable intervention. Comparing the effectiveness of stroboscopic training across different combat sports would help identify sport-specific adaptations and optimize training protocols for each discipline.

Third, future studies should investigate the effects of stroboscopic training on male boxers to determine whether the findings of this study are gender-specific. Male and female boxers differ in physiological characteristics (e.g., muscle mass, reaction time) and competitive styles, which may influence the response to stroboscopic training. A direct comparison between male and female boxers would provide a more comprehensive understanding of the training’s effectiveness across genders.

Fourth, research should explore the combination of stroboscopic training with other visual training methods (e.g., virtual reality training, eye-tracking training) to determine whether synergistic effects can be achieved. Virtual reality training, for example, can simulate realistic competitive environments, while stroboscopic training enhances visual-motor integration—combining these methods may yield greater improvements in performance than either method alone.

Fifth, future studies should investigate the effects of stroboscopic training on young female boxers (e.g., adolescent athletes) to determine whether early intervention can enhance the development of visual-motor skills and prevent performance plateaus. The nervous system is more plastic during adolescence, making this a critical period for skill development—stroboscopic training may be particularly effective in this population.

Finally, research should focus on translating the findings of this study into practical training guidelines for coaches and athletes. Developing standardized stroboscopic training protocols tailored to the specific needs of female boxers would ensure that the benefits of this training method are accessible to a wider range of practitioners. Additionally, conducting educational workshops for coaches on the implementation and monitoring of stroboscopic training would help promote the adoption of evidence-based training practices in the boxing community.

### 4.7 Conclusion

In conclusion, this study demonstrates that a six-week stroboscopic training program significantly improves key visual performance skills (EHC, RT, GNG, PS, MOT) and boxing performance (punching accuracy and defensive effectiveness) in female amateur boxers. The improvements observed in the experimental group were retained four weeks post-intervention, albeit with a slight decline, indicating the need for periodic booster sessions to maintain long-term effects. These findings extend the existing literature by highlighting the effectiveness of stroboscopic training in a previously understudied population (female boxers) and demonstrating transfer of training effects to actual competitive performance.

The practical implications of this study are clear: stroboscopic training is a feasible and effective intervention that can be integrated into existing training programs to enhance the visual-motor skills and competitive performance of female boxers. By prioritizing progressive training protocols and sport-specific drills, coaches can maximize the benefits of this training method for both offensive and defensive performance. While the study has several limitations, the findings provide a strong foundation for future research exploring the optimal implementation of stroboscopic training in combat sports.

Overall, this study contributes to the growing body of evidence supporting the use of stroboscopic training as a tool to enhance athletic performance and provides valuable insights for coaches, athletes, and sports scientists involved in female boxing. As the field of sports vision continues to evolve, stroboscopic training is likely to become an increasingly important component of evidence-based training programs for combat sports athletes.

## Data Availability

The raw data supporting the conclusions of this article will be made available by the authors, without undue reservation.

## References

[B1] Appelbaum L. G. Cain M. S. Schroeder J. E. Darling E. F. Mitroff S. R. (2012). Stroboscopic visual training improves information encoding in short-term memory. Atten. Percept. Psy. 74, 1681–1691. doi: 10.3758/s13414-012-0344-6 22810559

[B2] Appelbaum L. G. Erickson G. (2018). Sports vision training: A review of the state-of-the-art in digital training techniques. Int. Rev. Sport. Exerc. Psychol. 11, 160–189. doi: 10.1080/1750984X.2016.1266376 37339054

[B3] Appelbaum L. G. Lu Y. Khanna R. Detwiler K. R. (2016). The effects of sports vision training on sensorimotor abilities in collegiate softball athletes. Athl. Train. Sports. Health Care 8, 154–163. doi: 10.3928/19425864-20160314-01

[B4] Appelbaum L. G. Schroeder J. E. Cain M. S. Mitroff S. R. (2011). Improved visual cognition through stroboscopic training. Front. Psychol. 2, 276. doi: 10.3389/fpsyg.2011.00276 22059078 PMC3203550

[B5] Buscemi A. Mondelli F. Biagini I. Gueli S. D’Agostino A. Coco M. (2024). Role of sport vision in performance: Systematic review. J. Funct. Morphol. Kinesiol. 9, 92. doi: 10.3390/jfmk9020092 38921628 PMC11204951

[B6] Davis P. Benson P. R. Pitty J. D. Connorton A. J. Waldock R. (2015). The activity profile of elite male amateur boxing. Int. J. Sports. Physiol. Perform. 10, 53–57. doi: 10.1123/ijspp.2013-0474 24912199

[B7] Davis P. Benson P. R. Waldock R. Connorton A. J. (2016). Performance analysis of elite female amateur boxers and comparison with their male counterparts. Int. J. Sports. Physiol. Perform. 11, 55–60. doi: 10.1123/ijspp.2014-0133 25933441

[B8] Davis P. Wittekind A. Beneke R. (2013). Amateur boxing: Activity profile of winners and losers. Int. J. Sports. Physiol. Perform. 8, 84–92. doi: 10.1123/ijspp.8.1.84 22869640

[B9] Dendy D. James C. R. Brooks T. Lierly M. Riley N. Munger L. (2025). Case study: Effect of stroboscopic vision training during a softball season. Top. Exerc. Sci. Kinesiol. 6, 3. Available online at: https://oasis.library.unlv.edu/scholarship_kin/vol6/iss1/3.

[B10] Dunn E. C. Humberstone C. E. Iredale K. F. Martin D. T. Blazevich A. J. (2017). Human behaviours associated with dominance in elite amateur boxing bouts: A comparison of winners and losers under the ten point must system. PloS One 12, e0188675. doi: 10.1371/journal.pone.0188675 29287064 PMC5747423

[B11] Ellison P. Jones C. Sparks S. A. Murphy P. N. Page R. M. Carnegie E. . (2020). The effect of stroboscopic visual training on eye-hand coordination. Sport. Sci. Health 16, 1–10. doi: 10.1007/s11332-019-00615-4 30311153

[B12] Erickson G. B. (2018). Optimizing visual performance for sport. Adv. Ophthalmol. Optom. 3, 1–19. doi: 10.1016/j.yaoo.2018.05.001 38826717

[B13] Erickson G. B. Citek K. Cove M. Wilczek J. Linster C. Bjarnason B. . (2011). Reliability of a computer-based system for measuring visual performance skills. Optometry 82, 528–542. doi: 10.1016/j.optm.2011.01.012 21705283

[B14] Faul F. Erdfelder E. Lang A. G. Buchner A. (2007). G*Power 3: A flexible statistical power analysis program for the social, behavioral, and biomedical sciences. Behav. Res. Methods 39, 175–191. doi: 10.3758/BF03193146 17695343

[B15] Giustino V. Bonaventura R. E. Messina G. Patti A. Pillitteri G. Pajaujiene S. . (2024). Acute effects of prismatic adaptation on penalty kick accuracy and postural control in young soccer players: A pilot study. Heliyon 10, e30515. doi: 10.1016/j.heliyon.2024.e30515 38742074 PMC11089356

[B16] Hülsdünker T. Rentz C. Ruhnow D. Käsbauer H. Strüder H. K. Mierau A. (2019). The effect of 4-week stroboscopic training on visual function and sport-specific visuomotor performance in top-level badminton players. Int. J. Sports. Physiol. Perform. 14, 343–350. doi: 10.1123/ijspp.2018-0302 30160560

[B17] Hülsdünker T. Gunasekara N. Mierau A. (2021). Short- and long-term stroboscopic training effects on visuomotor performance in elite youth sports. Part 1: Reaction and behavior. Med. Sci. Sports. Exerc. 53, 960–972. doi: 10.1249/MSS.0000000000002541 33060548

[B18] Jones C. Carnegie E. Ellison P. (2016). “ The effect of stroboscopic vision training on eye-hand coordination”, in: British Psychological Society (BPS) Division of Sport & Exercise Science Conference.

[B19] Knöllner A. Memmert D. von Lehe M. Jungilligens J. Scharfen H. E. (2022). Specific relations of visual skills and executive functions in elite soccer players. Front. Psychol. 13, 960092. doi: 10.3389/fpsyg.2022.960092 36092125 PMC9454603

[B20] Laby D. M. Kirschen D. G. Pantall P. (2011). The visual function of Olympic-level athletes—An initial report. Eye. Contact. Lens. 37, 116–122. doi: 10.1097/ICL.0b013e31820c5002 21378577

[B21] Lee H. Han S. Page G. Bruening D. A. Seeley M. K. Hopkins J. T. (2022a). Effects of balance training with stroboscopic glasses on postural control in chronic ankle instability patients. Scand. J. Med. Sci. Sports. 32, 576–587. doi: 10.1111/sms.14098 34775656

[B22] Lee J. S. Liu Y. H. Chen W. M. Lin K. K. Chang S. T. Lim A. Y. . (2022b). Association of sports vision with age, gender, and static visual acuity among nonathletic population. Taiwan. J. Ophthalmol. 12, 53–60. doi: 10.4103/tjo.tjo_60_20 35399972 PMC8988984

[B23] Liu S. Ferris L. M. Hilbig S. Asamoa E. LaRue J. L. Lyon D. . (2020). Dynamic vision training transfers positively to batting practice performance among collegiate baseball batters. Psychol. Sport. Exerc. 51, 101759. doi: 10.1016/j.psychsport.2020.101759 38826717

[B24] Millard L. Shaw I. Breukelman G. J. Shaw B. S. (2020). Factors affecting vision and visio-spatial intelligence (VSI) in sport: A review of the literature. Asian J. Sports. Med. 11, e101670. doi: 10.5812/asjsm.101670

[B25] Müller S. Beseler B. Morris-Binelli K. Mesagno C. (2024). Temporal samples of visual information guides skilled interception. Front. Psychol. 15, 1328991. doi: 10.3389/fpsyg.2024.1328991 38469214 PMC10925617

[B26] Palmer T. Coutts A. J. Fransen J. (2022). An exploratory study on the effect of a four-week stroboscopic vision training program on soccer dribbling performance. Braz. J. Mot. Behav. 16, 254–265. doi: 10.20338/bjmb.v16i3.310

[B27] Poltavski D. Biberdorf D. (2015). The role of visual perception measures used in sports vision programmes in predicting actual game performance in Division I collegiate hockey players. J. Sports. Sci. 33, 597–608. doi: 10.1080/02640414.2014.951952 25142869

[B28] Poltavski D. Biberdorf D. Praus Poltavski C. (2021). Which comes first in sports vision training: The software or the hardware update? Utility of electrophysiological measures in monitoring specialized visual training in youth athletes. Front. Hum. Neurosci. 15, 732303. doi: 10.3389/fnhum.2021.732303 34690722 PMC8527177

[B29] Ripoll H. Kerlirzin Y. Stein J. F. Reine B. (1995). Analysis of information processing, decision making, and visual strategies in complex problem solving sport situations. Hum. Mov. Sci. 14, 325–349. doi: 10.1016/0167-9457(95)00019-O

[B30] Shabeb A. A. S. (2017). The impact of qualitative visual vision trainings on the concentration of attention and precision of counter-attack for boxing players. Int. Sci. J. Phys. Educ. Sport. Sci. 5, 77–96. doi: 10.21608/isjpes.2017.60377

[B31] Stanley E. Thomson E. Smith G. Lamb K. L. (2018). An analysis of the three-dimensional kinetics and kinematics of maximal effort punches among amateur boxers. Int. J. Perform. Anal. Sport. 18, 835–854. doi: 10.1080/24748668.2018.1525651 37339054

[B32] Tarasi L. Turrini S. Sel A. Avenanti A. Romei V. (2024). Cortico-cortical paired-associative stimulation to investigate the plasticity of cortico-cortical visual networks in humans. Curr. Opin. Behav. Sci. 56, 101359. doi: 10.1016/j.cobeha.2024.101359 38826717

[B33] Thomson E. Lamb K. Nicholas C. (2013). The development of a reliable amateur boxing performance analysis template. J. Sports. Sci. 31, 516–528. doi: 10.1080/02640414.2012.738922 23121380

[B34] Wang L. Krasich K. Bel-Bahar T. Hughes L. Mitroff S. R. Appelbaum L. G. (2015). Mapping the structure of perceptual and visual-motor abilities in healthy young adults. Acta Psychol. 157, 74–84. doi: 10.1016/j.actpsy.2015.02.005 25747573

[B35] World Medical Association (2013). World Medical Association Declaration of Helsinki: Ethical principles for medical research involving human subjects. JAMA 310, 2191–2194. doi: 10.1001/jama.2013.281053 24141714

[B36] Wu R. Yang Q. Cui W. Gao D. Luo Y. Wang D. (2024). Relationship between visual ability assessment and punch performance in competition in male amateur boxers. Front. Physiol. 15, 1429554. doi: 10.3389/fphys.2024.1429554 39081778 PMC11286555

[B37] Zwierko T. Jedziniak W. Domaradzki J. Zwierko M. Opolska M. Lubiński W. (2024). Electrophysiological evidence of stroboscopic training in elite handball players: Visual evoked potentials study. J. Hum. Kinet. 90, 57–69. doi: 10.5114/jhk/169443 38380298 PMC10875695

[B38] Zwierko M. Jedziniak W. Popowczak M. Rokita A. (2023). Effects of in-situ stroboscopic training on visual, visuomotor and reactive agility in youth volleyball players. PeerJ 11, e15213. doi: 10.7717/peerj.15213 37250711 PMC10211363

